# How Do Russians Perceive and Justify the Status Quo: Insights From Adapting the System Justification Scales

**DOI:** 10.3389/fpsyg.2021.717838

**Published:** 2021-10-21

**Authors:** Elena Agadullina, Alexander Ivanov, Irena Sarieva

**Affiliations:** School of Psychology, HSE University, Moscow, Russia

**Keywords:** system justification, general system justification, political system justification, economic system justification, gender system justification, scale

## Abstract

This paper aims to adapt the system justification scales (general, political, economic, and gender) for the Russian socio-cultural context and to carry out their psychometric analysis. In Study 1 (*N* = 450), the original items from four system justification scales on a Russian sample were tested. The results suggested that none of the original scales worked properly in Russia and all of them demonstrated weak fit indices. In Study 2 (*N* = 553), culturally specific grounds for system justification in the Russian context were highlighted and tested. The results of this study confirmed that all the adapted scales demonstrated a good fit to the data. In Study 3 (*N* = 1,011), the invariance of system justification scales and the convergent and discriminant validity of the scales was tested. The results confirmed that the general, political, and economic system justification scales demonstrated full invariance, while the gender system justification scale showed partial invariance for multigroup comparison. All the scales convergent and discriminant validity, which leads to consider them as an effective tool for measuring system justification in various types of social relationships in Russia. The role of socio-cultural characteristics in justifying the status quo is discussed.

## Introduction

The System Justification Theory (SJT) was proposed by John Jost and Mahzarin Banaji in 1994. They suggested that people are motivated to justify the status quo by sharing prejudices, stereotypes, and ideologies that legitimize existing social relationships in society and rationalize them as essential and fair (Jost and Banaji, 1994). According to SJT, people are often not able unable to assess social inequality objectively because it is masked or underestimated by stereotypes with ambivalent content (which includes both positive features of low-status groups and negative features of high-status groups) (e.g., Durante et al., 2017) or different ideologies (e.g., conservatism or nationalism) (Jost, 2019).

Underestimation of existing inequality leads to both perceiving an existing system as fair and legitimate and to showing outgroup favoritism toward high-status groups. Previous studies have confirmed that various low-status groups—for example, sexual minorities (Jost et al., 2004; Bahamondes-Correa, 2016), the poor (Harvey and Bourhis, 2013; Horwitz and Dovidio, 2017), overweight people (Rudman et al., 2002), and migrants, ethnic minorities or low-status racial groups (Ashburn–Nardo et al., 2003)—demonstrated more positive attitudes toward an outgroup than to an ingroup.

Regarding the perception of system as fair and legitimate, researchers have found that SJ is directly related to an unwillingness to change the system, the lack of protest-related activity in the face of noticeable inequality (Osborne and Sibley, 2013), support for collective actions that are aimed at preserving the status quo (Osborne et al., 2019), the presence of a negative attitude toward corruption in line with an unwillingness to fight against it (Tan et al., 2016), and so on. In other words, system justification reinforces existing social mechanisms and impedes social change. Jost (2019) posited that to fully understand the motivation and belief of various groups in regard to social change, SJT is as important as Social identity theory (Tajfel and Turner, 1986). Considering the increasing demand for social change (for example, in political and economic relationships or gender-related politics), research within the framework of SJT is becoming more and more relevant.

## System Justification Domains and Scales

During the last 25 years, the notion of “system justification” has been actively used in social sciences for a more thorough understanding of societal functioning, predicting collective behavior in the social, economic, and political context. Jost and colleagues assumed that SJ motivation can be applied to different life domains and relationship types: social (general), economic, gender, and political (Jost, 2019). There are scales for measuring SJ in each domain.

The economic SJ scale was developed in 2000 (Jost and Thompson, 2000). This scale measures the belief that economic inequality is natural, inevitable, and legitimate, while a person's income is always deserved. The scale consists of 17 statements (e.g., “If people work hard, they almost always get what they want”). Previous studies have shown that economic SJ is associated with an opposition to economic equality (Jost and Thompson, 2000) along with a muted emotional response to inequality (Goudarzi et al., 2020), ethnocentrism (Jost and Thompson, 2000), and political conservatism (Jost and Thompson, 2000; Jost et al., 2017). In a broader context, economic SJ is associated with less negative attitudes toward economic inequality (Goudarzi et al., 2020). Therefore, economic SJ can be one of the factors leading to the rapid growth of economic inequality that was recently observed in the world (Wilkinson and Pickett, 2017).

The general SJ scale was introduced in 2003 (Kay and Jost, 2003). It includes eight statements related to the perception of the fairness and legitimacy of the existing system, quality of life, and the chance to achieve happiness and wealth (e.g., “In general, you find society to be fair”). Researchers have shown that general SJ is closely related to right-wing authoritarianism (Azevedo et al., 2017), social dominance orientation (Jylhä and Akrami, 2015), a just world belief (Kelemen et al., 2014), nationalism and patriotism (Carter et al., 2011), and political conservatism (Jost et al., 2008), as well as economic and social conservatism (Everett, 2013). All these constructs and ideologies support traditional values, hierarchy, and justification of group dominance from a biological or a social perspective. In the context of behavior, justifying the system was associated with voting for a more conservative politician and supporting right-wing ideology (for example, in developed capitalist countries such as the United States) (Azevedo et al., 2017). In contrast, it was not associated with political orientations but linked to being enrolled in ruling party among Eastern Europeans (Szabó and Lönnqvist, 2021).

The gender SJ scale was published in 2005 (Jost and Kay, 2005). It includes eight statements (e.g., “In general, relations between men and women are fair”) which describe gender differences as inherent and inevitable, as well as the fairness of traditional gender roles. Gender SJ is associated with sexism (Howard et al., 2020) and gender essentialism (Brescoll et al., 2013). All these constructs outline crucial differences between men and women and support traditional gender roles, which serves a system-justifying function. High gender system justification is negatively associated with supporting women in politics (Azevedo et al., 2017). It also maintains gender inequality in society (Napier et al., 2010) and reinforces the higher status of men (Glick et al., 2000).

The political SJ scale was developed in 2010 (Jost et al., 2010). It measures belief in the fairness of the existing political system, whatever that happens to be, and beliefs that the system in general and the authorities, in particular, are willing to fulfill citizens' needs and fight for their interests. This scale includes six statements (e.g., “In general, the American political system operates as it should”). Previous studies have found that political SJ is closely related to political self-efficacy (Osborne et al., 2015) and readiness to protest (Osborne et al., 2019). Justifying political system also promoted greater trust in government and decreased willingness to deal with complex political issues (Shepherd and Kay, 2012).

Most studies of various types of SJ have been conducted in the USA using the original scales proposed by Jost et al., though there have been numerous attempts to adapt the SJ scales to different socio-cultural contexts. In particular, there are Finnish (Vainio et al., 2014), German (Ullrich and Cohrs, 2007), Polish (Cichocka et al., 2015), and Hungarian (van der Toorn et al., 2010) versions of the general SJ scale. A common feature of these adaptations is that they do not provide complete statistical information about the adapted scale (except Cronbach's alpha) and outline mere validity evidence. As a result, it is rather difficult to assess the quality of the adapted scales, as well as their suitability for further use.

The main aim of this paper is to adapt the SJ scales for the Russian socio-cultural context and to carry out a thorough psychometric analysis of them.

## Social Context of the Study

Most studies of SJ have been conducted in Western, Educated, Industrialized, Rich, and Democratic (WEIRD) countries (Henrich et al., 2010) with governments with relatively stable long-term histories. Much less attention has been paid to non-typical WEIRD countries and to countries that have experienced dramatic changes in the social order and governmental and ideological systems.

Previous studies have shown that in Western countries the level of system justification is higher than, for example, in Hungary and Poland (van der Toorn et al., 2010; Cichocka and Jost, 2014)—Eastern European countries which have been socialist for a long time. Szabó and Lönnqvist (2021) demonstrated that during the past years system derogation was stronger than system justification in Hungary (Szabó and Lönnqvist, 2021). Kelemen et al. (2014) explained the low level of SJ among Hungarians by a still-existing nostalgia for socialist equality. In the socialist period equality was more important than individual merit but in post-socialist time merit become more important for social relationship. Such changes could be perceived as unjust by people who were used to the previous social order. Cichocka and Jost (2014) also connected the system derogation with the critical system transformation that communist societies underwent at the end of the 20th Century. In their opinion, people in post-communist countries were disillusioned by the previous system and had unrealistically high expectations of the new, capitalist system. As those expectations have not been fulfilled, people have lost trust in any system at all. These examples suggest that broad historical and socio-cultural context can play a crucial role in the manifestation of SJ. Russia is one of the countries that went through major social and political transformation. The changes and social upheavals that occurred after the collapse of the Soviet Union created a unique social context for the perception of the status quo that was established in the years that followed. Nowadays, Russia is a relatively young hybrid authoritarian regime with general elections (Hale, 2010), where large percentage of the population was born and grew up in the Soviet Union and remember well the structure of social and political life in those times. Studying SJ in Russia could become an interesting unique case and enrich the understanding of how SJ works.

In modern Russia, there are several signs that may indicate a prevalence among the population of a willingness to justify the existing status quo. For example, there is a high level of corruption in Russia (Transparency International, 2019); however, only 39% of the population believes that this is a really serious problem (Levada Center, 2020c). A similar situation can be seen around the perception of inequality; only 27% of the population believe that inequitable distribution of income is a serious problem for Russia (Levada Center, 2020c). World Bank data, however, shows that Russia has a higher level of economic inequality compared to the developed European countries (The World Bank, 2018).

Also, there is a very low level of protest-related activity in Russia. According to sociological polls, 72% of Russians are not ready or willing to take part in any collective actions against the decline of the standard of living or for the defense of human rights (Levada Center, 2020d). Even more people, 78%, are not ready or willing to take part in a political protest (ibid). At the same time, trust in political institutions (government, the state duma, political parties) is quite low: only from 22 to 38% of the population trust these structures (Levada Center, 2020f).

Approbation of the SJ scales would be a real benefit for Russia, since it would help researchers to understand whether the perception of various social problems and the Russian people's level of political activity relate to their willingness to justify the existing status quo. In addition, the adaptation of the scales in a new socio-cultural context can provide new data on the role of the cultural characteristics in justifying the system, which will generally contribute to the development of the SJT. Finally, in current times when the questions of inequality in different areas (gender, economic, ethnic, and political) are still on the cutting edge of top social and scientific discussions it is crucially important to have a verified instrument to reliably measure the level of justifying beliefs in the context of various social processes.

## Study 1

The main purpose of Study 1 was to test the original items from four SJ scales on a Russian sample. For this purpose, all items were translated into Russian and back translated into English by independent experts with an advanced language level. All discrepancies in translation were discussed with experts to formulate the final version of the scales in Russian.

### Method

#### Sample

For this online study, 450 participants were recruited through Yandex.Toloka (the Russian equivalent of Amazon's Mechanical Turk) and received a participation fee. The sample was stratified based on sex and age. A detailed description of the sample is presented in Table 1.

**Table 1:** The description of the studies samples.

	**Study 1 (*****N*** **= 450)**		**Study 2 (*****N*** **= 553)**		**Study 3 (*****N*** **= 1,011)**	
	***N***	**%**	***N***	**%**	***N***	**%**
**Sex**						
Male	231	51	280	51	485	48
Female	219	49	273	49	526	52
**Age**	M = 34.18, *SD* = 10.66		M = 34.51, *SD* = 10.98		M = 35.10, *SD* = 11.94	
**Education**						
Elementary school			3	<1	2	1
High school	34	7	58	10	91	9
Vocational school (technical secondary school)	115	25	120	22	226	22
Higher education (students)	61	15	77	14	123	12
Higher education (completed)	240	53	295	53	569	56
**Religion**						
No religion	211	47	257	46	524	52
Orthodox	215	48	265	48	445	44
Muslim	4	1	8	1	25	2
Other Christians (Catholics, protestants)	7	2	8	1	6	1
Other non-Christians (Buddhists, hindus)	4	1	10	2	11	1
Other or missing	13	2	5	1	0	0
**Ethnicity**						
Russian	417	93	505	91	934	92
Tatar	9	2	14	3	42	4
Ukrainian	6	1	6	1	15	1
Belarusian	1	1	1	<1	4	<1
Jew	3	1	0	0	2	<1
Other or missing	14	2	14	3	14	<1
**Type of city (thousand residents)**						
> 100	97	22	118	22	137	13
100–250	32	7	51	9	61	6
250–500	64	14	85	15	99	10
500–1,000	80	18	83	15	100	10
<1,000 (including Moscow and St Petersburg)	177	39	216	39	614	61
**Political self-placement**						
Left	220	49	272	49	488	49
Centrist	146	32	187	34	369	37
Right	84	19	94	17	151	14
**Socio-economic status**						
Low	217	48	276	50	444	44
Middle	151	34	175	32	339	34
High	82	18	102	18	225	22
**Religiosity**						
Religious	145	32	167	30	269	27
Non-religious	215	48	278	50	532	53
Difficult to identify	90	20	108	20	210	20

#### Procedure and Measures

The study was conducted in April 2020. After providing the informed consent and receiving detailed instructions, participants completed a questionnaire (the raw data for Study 1 can be found on the OSF: https://osf.io/3wxge/?view_only=353fa3a12dca45a18c68cc003bd36eb5). On the first page, participants rated the general SJ scale, which included eight items (e.g., “Russia is the best country to live in”) (α = 0.88, M = 3.84, SD = 1.74). On the second page, participants were randomly presented with either the political, gender or economic SJ scale. The political SJ scale included six items (e.g., “The Russian political system is the best in the world”) (*n* = 164; α = 0.71, M = 4.25, SD = 1.53); the gender SJ scale included eight items (e.g., “In general, gender relations are fair”) (*n* = 112; α = 0.76, M = 5.06, SD = 1.37); and the economic SJ scale consisted of 17 items (e.g., “It is virtually impossible to eliminate poverty”) (*n* = 174; α = 0.75, M = 4.70, SD = 0.93). Participants rated all items on the scale from 1 (*disagree strongly*) to 9 (*agree strongly*). On the last page, participants answered socio-demographic questions (sex, age, education level, religiosity, ethnicity, socio-economic status, political self-placement, and city of residence).

### Results and Discussion

Confirmatory factor analysis (CFA) was carried out for each scale to assess how well the data fitted the expected theoretical model (Rosseel, 2012). We used the robust maximum likelihood (MLR) method that is effective in occurrences of data non-normality (Maydeu-Olivares, 2017). The following fit indices were used to evaluate the fit of a model to the data: the ratio of chi-square to degrees of freedom (χ2/*df*), the root mean square error of approximation (RMSEA), the comparative fit index (CFI), the Tucker-Lewis index (TLI), and the standardized root mean square residual (SRMR). The values of RMSEA < 0.06; SRMR ≤ 0.08; TLI ≥0.95 (Schreiber et al., 2006); CFI ≥0.95 and χ^2^/df <3 (Hu and Bentler, 1999) were considered as indicators of good fit.

Before data processing, all reverse items were recoded. Table 2 shows the results of CFA and demonstrates that all the adapted scales had unacceptable fit indices. The economic SJ scale demonstrated the worst fit to the data.

**Table 2:** CFA one-factor goodness of fit indicators of the Russian versions of SJ scales.

**Model**	**χ^**2**^**	***df***	**CFI**	**TLI**	**SRMR**	**RMSEA**	**95% CI RMSEA**
General SJ	228.528	20	0.889	0.845	0.069	0.124	[0.111, 0.136]
Political SJ	81.216	9	0.646	0.410	0.110	0.208	[0.170, 00.248]
Gender SJ	57.572	20	0.881	0.833	0.069	0.102	[0.075, 0.130]
Economic SJ	493.792	119	0.427	0.345	0.140	0.130	[0.119, 0.141]

There are at least two possible reasons for the results obtained. The first reason might relate to the reverse-scored items in the original scales. The results demonstrated that reverse-scored items in the general (3 and 7), political (4 and 6), and gender (3 and 7) SJ scales had low or negative factor loadings. This situation is common in many psychometric scales, as the combination of reverse-scored and positive items tends to provide difficulties in understanding for respondents and additional sources of variance that jeopardize the unidimensionality of the test (Salazar et al., 2015; Suárez-Alvarez et al., 2018).

The second reason might relate to different understandings of the original items by Russian respondents. For example, the item “The multi-party electoral system is democracy at its best” could be misunderstood by Russians because while Russia *de jure* has a multi-party system, almost 76% of the seats in the current State Duma are held by the United Russia party (colloquially known as “the party of power”) (http://duma.gov.ru). This could lead to perception of the political system as a *de facto* one-party system. This perception may be familiar to many Russians, as the Soviet Union also had a one-party system.

Items from the economic SJ scale that are relevant for Western economic and political discourse may come into dissonance with the conviction of Russians that the government should care about the economic well-being of citizens. According to the sociologist Lev Gudkov, Russians' ideas about the interaction between the government and citizens could be characterized as follows: “We don't trust the authorities, but believe that government must care for a decent life for citizens—this is a very stable discrepancy dating back to the times of Soviet paternalism” (Levada Center, 2018b, 2020e). Thus, it can be concluded that the SJ scales require greater cultural adaptation to the Russian context.

## Study 2

The purpose of this study was to highlight and test the culturally specific grounds for SJ in the Russian context. Several bases that may be employed as a rationale of SJ were identified.

First, the belief that the president and the ministry introduce good policies which end up not making any positive change due to corrupt or unqualified local authorities. This pattern of lay thinking is reflected well in a popular Russian saying: “Good Tsar but bad boyars.” Opinion polls regularly demonstrate high levels of support for and legitimization of the president, in comparison with a very low level of support for local authorities in Russia (Levada Center, 2020f).

Second, the term “stability” has a positive connotation in Russia. In societal discourse, there is a widespread belief that stability, rather than social change, is beneficial to economic growth. According to polls of Russians, 63% believe that stability and 66% believe that order must be priorities in the country's development, while only 30% of respondents believe that the country needs some kind of change (WCIOM, 2016).

Third, the belief that Russia has a unique way of development based on its history and traditions is widespread. Therefore, the practices and actions of foreign governments, along with societal developments and ideologies that have proven to be effective in other countries, are seen as inapplicable in Russia (Levada Center, 2014).

Finally, the current political context and trends provide a new basis for justification. Sanctions from some western countries introduced in 2014 became a popular topic in lay and media discourse (Kazun, 2016). Despite rising inflation, the media discourse supports the idea that these sanctions do not impact the welfare of Russians. According to recent social surveys, 67% of Russians agree with this (Levada Center, 2020b).

Using these specifics for Russian socio-cultural context bases for SJ, some of the original statements for SJ scales were reformulated and formulated some new ones. As a result, 11 items were formulated for the general SJ scale, 13 for the political SJ scale, 11 for the economic SJ scale, and 10 for the gender SJ scale (a complete list of items in each scale is presented in the Appendix on OSF). In the next step, all the items to select those that best fitted the various SJ scales in the Russian socio-cultural context were tested.

### Method

#### Participants

Again using Yandex.Toloka, 553 participants were recruited to this online study in return for a participation fee. The sample was stratified based on sex and age. A detailed description of the sample is shown in Table 1.

#### Measures and Procedure

The study was conducted in May 2020 (the raw data for Study 2 can be found on OSF). After providing the informed consent, respondents completed a questionnaire that included two parts. In the first part, respondents completed the SJ scales: general (14 items (e.g., “In Russia today, life does not need any significant changes”); political [13 items (e.g., “In Russia today, the authorities in general can be trusted”)]; economic (11 items (e.g., “In Russia today, salaries are fair and adequate”); and gender (10 items (e.g., “In Russia today, relations between men and women are what they are supposed to be”). Participants rated all items on a scale from 1 (*disagree strongly*) to 9 (*agree strongly*). In the second part, participants answered the same socio-demographic questions that were asked in Study 1.

### Results and Discussion

To fulfill the aim of the study, a series of CFAs was conducted. First, the scales with all selected items to assess the contribution of each item to the scale were tested. Table 3 shows the results of the analyses of initial models and demonstrates a poor fit to the data for all models.

**Table 3:** Study 2 CFA summary.

**Model**		**χ2**	***df***	**CFI**	**TLI**	**SRMR**	**RMSEA**	**95% CI RMSEA**
General SJ	Initial model	186.474	44	0.940	0.924	0.045	0.077	[0.067, 0.092]
	Adjusted model	5.838	6	0.999	0.998	0.010	0.017	[0.000, 0.052]
Political SJ	Initial model	410.518	65	0.918	0.902	0.051	0.098	[0.090, 0.106]
	Adjusted model	44.960	27	0.993	0.991	0.015	0.035	[0.019, 0.049]
Gender SJ	Initial model	121.645	35	0.913	0.888	0.049	0.067	[0.056, 0.078]
	Adjusted model	19.930	9	0.981	0.968	0.026	0.047	[0.024, 0.069]
Economic SJ	Initial model	238.051	44	0.831	0.789	0.063	0.089	[0.078, 0.101]
	Adjusted model	19.880	9	0.981	0.968	0.029	0.047	[0.022, 0.071]

Analysis of items within the scales showed that for the general SJ scale, items 1, 2, 8, 9, 10, and 11 demonstrated low factor loadings and level of explained variance (< 0.45) in comparison with other items. Moreover, item 1 had covariance with item 2, 3, and 5, items 9–10 and items 4–11 demonstrated strong covariance. For the political SJ scale, items 8, 10, and 13 demonstrated low factor loadings and level of explained variance (< 0.55); additionally, items 10–11 and 8–13 demonstrated strong covariance. For the economics SJ scale, items 1, 2, and 10 demonstrated low factor loadings and level of explained variance and items 4 and 11 had strong covariance with other items. Finally, for the gender SJ scale, items 1, 2, and 4 had low factor loadings and explained variance, and item 10 had strong covariations with other items. All items with a low factor loading and strong covariance were removed from the scales. As a result, the short version of the general SJ scale included five items, the political SJ scale had nine items, and the economics and gender SJ scales each included six items.

**Figure 1:**
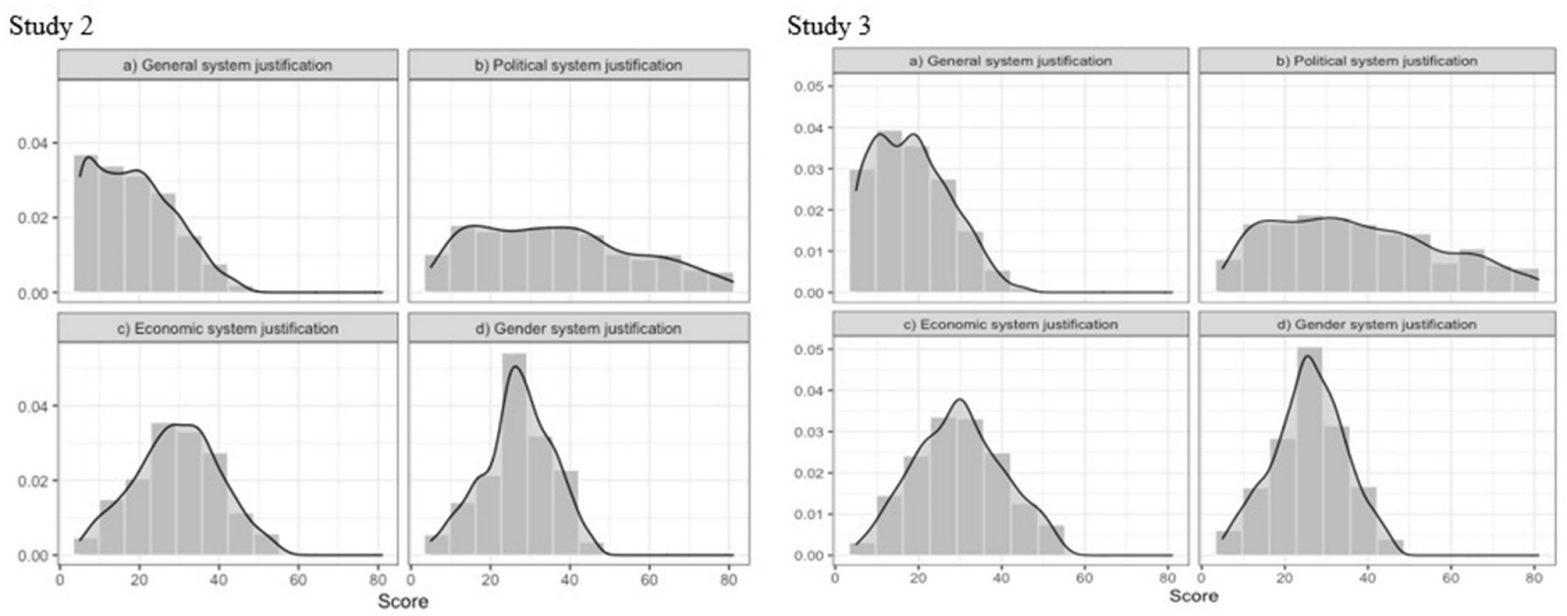
Distribution of the total score of SJ scales from Study 2 and Study 3.

Repeated CFAs were conducted for the short versions of the scales and demonstrated a good fit to data for all scales (see Table 3). Table 4 shows the descriptive statistics and internal consistency for the adjusted models. Distribution of the total score for all scales is shown in Figure 1. The distribution of the total score for the economic and gender SJ is close to symmetric (Figures 1C,D). At the same time, for the general SJ scale, the distribution is significantly right skewed (Figures 1A,B), indicating the low level of this type of justification in the sample. An identical tendency was observed in other post-communist societies. Cichocka and Jost (2014) demonstrated that in such societies, the level of general and political SJ is significantly lower than in capitalist ones. The distribution of the total score of the political SJ scale does not demonstrate any pronounced bias and indicates that the sample equally includes people with different levels of political SJ.

**Table 4:** Descriptive statistics for SJ scales in Study 2 (*n* = 553) and Study 3 (*n* = 1,011).

**Scale**	**Mean**	**SD**	**Median**	**Min**	**Max**	**α**
General SJ	3.70/3.69	2.02/1.83	3.60/3.60	1/1	9/9	0.92/0.89
Political SJ	4.09/4.15	2.17/2.16	3.88/3.89	1/1	9/9	0.96/0.95
Gender SJ	4.97/5.16	1.69/1.73	5.00/5.14	1/1	9/9	0.80/0.76
Economic SJ	4.91/4.95	1.78/1.79	5.00/5.00	1/1	9/9	0.78/0.79

*Note. Study 2 before the slanted line, Study 3 after the slanted line*.

In general, the results of Study 2 show that items that are more adapted to the cultural context form well-balanced scales of SJ.

## Study 3

The aim of Study 3 was 2-fold: to retest the factor structure of the SJ scales obtained in Study 2, and to collect validity evidence for SJ scales. To fulfill the second aim, we measured various ideological beliefs and attitudes, which also serve a system-justifying function and preserve the status quo in different types of social relationships were measured. As previous studies have shown economic SJ is closely related to opposition to economic equality (Jost and Thompson, 2000) and as well as general SJ is associated with right-wing authoritarianism (Azevedo et al., 2017), social dominance orientation (Jylhä and Akrami, 2015), and a just world belief (Kelemen et al., 2014). Gender SJ is related to sexism (Howard et al., 2020) while political SJ is associated with political self-efficacy (Osborne et al., 2015) and readiness to protest (Osborne et al., 2019). The correlations between the tested scales and the specified variables will indicate the scales' convergent validity. In addition, previous studies demonstrated that openness to experiences was not related to SJ (Jost, 2019), therefore, it can be used to check the discriminant validity of the scales.

### Method

#### Sample

For Study 3, 1,011 participants were recruited using Yandex.Toloka platform and received a participation fee. The sample was stratified based on respondents' sex, age, and region of residence. A detailed description of the sample is shown in Table 1.

#### Measures

The following measures were used:

*System justification* (SJ) was measured by the four final scales from Study 2.

The *Right-wing authoritarianism* (RWA) scale (Altemeyer, 1998) in the Russian adaptation (Chertkova et al., 2017). The scale includes nine items (e.g., “The authorities should get rid of those who are for crucial changes in society”) that participants rated from 1 (*disagree strongly*) to 7 (*agree strongly*) (*a* = 0.88).

The “dominance” subscale from the *social dominance orientation* (SDO) scale (Pratto et al., 1994), using the Russian adaptation (Gulevich et al., 2018). The scale includes five items (e.g., “Some social groups are better than others”) that participants rated from 1 (*disagree strongly*) to 5 (*agree strongly*) (*a* = 0.78).

The *just-world belief* (JWB) scale (Dalbert, 1999) in the Russian adaptation (Nartova-Bochaver et al., 2013). The scale includes six items (e.g., “In general, life treats me fair”) that participants rated on a scale from 1 (*disagree strongly*) to 5 (*agree strongly*) (α = 0.80).

*Openness to experience* (OTE) was measured by a subscale of a Russian version of the Big Five personality inventory developed by Shchebetenko (2014). The subscale includes 12 adjectives describing a person, such as “creative” and “complex.” Participants rated each trait on a scale from 1 (*disagree strongly*) to 5 (*agree strongly*) according to the level they possess this trait (α = 0.80).

*The external efficacy subscale of the political self-efficacy* (PSE) measure, which includes three items (e.g., “The people in charge of government are willing to provide information on how political decisions are made”) that participants rated on a scale from 1 (*disagree strongly*) to 5 (*agree strongly*) (α = 0.89) (Sarieva, 2018).

*Readiness to protest* (RTP) was measured through agreement to participate in street protests. Respondents rated their level of agreement on a scale from 1 (*disagree strongly*) to 5 (*agree strongly*) (M = 2.55, *SD* = 1.18).

*Perceived economic inequality* (PEI) was measured through the level of agreement with the statement “There is economic inequality in Russia,” which participants rated on a scale from 1 (*disagree strongly*) to 7 (*agree strongly*) (M = 4.96, *SD* = 1.01).

*Base income support* (BI) was measured by the question “Do you agree that base income for everybody must be employed in Russia?” Participants rated their answers on a scale from 1 (*disagree strongly*) to 7 (*agree strongly*) (M = 5.13, *SD* = 1.95).

*Equal distribution support* (ED) was measured by the question “Do you agree that it is necessary to distribute resources in Russia more evenly between low and high-status groups?” Participants rated their answers on a scale from 1 (*disagree strongly*) to 7 (*agree strongly*) (M = 4.48, *SD* = 1.96).

*Ambivalent sexism* was measured by the Russian adaptation of the Ambivalent Sexism Inventory (Agadullina, 2018). The inventory includes 12 statements: six relating to hostile sexism (HS) (e.g., “women seek to gain power by gaining control over men”) and six relating to benevolent sexism (BS) (e.g., “women should be cherished and protected by men”). Participants rated all statements on a scale from 1 (*disagree strongly*) to 6 (*agree strongly*) (*a* = 0.87 and 0.90 for HS and BS, respectively).

*Religiosity* was measured by the question “Do you consider yourself be a religious person?” with three options for the answer (“yes,” “no,” and “difficult to identify”).

*Socio-economic status* measure included the question “How would you rate your income level?” The respondents had to choose one of the options from 1 (*there is not enough money even for food*) to 6 (*I can afford everything*) (for details, see Table 1). The first three options identified a low status, option four indicated middle-class status, and the last two options reflected a high socio-economic status.

#### Procedure

The study was conducted in December 2020. After providing the informed consent, respondents completed a questionnaire (the raw data for Study 3 can be found on OSF). On the first page, participants completed the general SJ scale and the RWA, SDO, JWB, and OTE scales. On the second page, they filled out the political SJ scale and the PSE, as well as the question about RTP. On the third page, participants completed the economic SJ scale and questions about PEI, BI, and ED. On the fourth page, participants completed the gender SJ scale, HS, and BS scales. On the last page, participants filled the same socio-demographics that was used in Studies 1 and 2.

### Results and Discussion

To re-test the scales identified in Study 2, series of CFAs were conducted. The results shown in Table 5 demonstrate that all the scales had a good fit to the data. The descriptive statistics and internal consistency coefficients for all scales are shown in Table 4, and the distribution of the total score of the scales is shown in Figure 1. The results show that all the trends identified in Study 2 related to scale consistency and the distribution of total scores were reproduced in Study 3.

**Table 5:** The results of CFAs for Study 3.

**Model**	**χ2**	***df***	**CFI**	**TLI**	**SRMR**	**RMSEA**	**95% CI RMSEA**
General SJ	17.30**	5	0.992	0.984	0.014	0.049	[0.031, 0.069]
Political SJ	106.44***	27	0.983	0.977	0.016	0.054	[0.045, 0.063]
Gender SJ	22.16***	5	0.978	0.956	0.028	0.058	[0.038, 0.081]
Economic SJ	26.29**	9	0.985	0.974	0.023	0.044	[0.027, 0.060]

** *p < 0.01*,

*** *p < 0.001*.

#### Multigroup Confirmatory Factor Analyses

To check whether the SJ scales had invariance for different groups, a multigroup confirmatory factor analysis (MCFA) was conducted using RStudio Semtools (Jorgensen et al., 2018) and lavaan (Rosseel, 2012) packages.

This analysis tests the configural (same structure), metric (same factor loadings), and scalar (same factor loadings and item intercepts) (Putnick and Bornstein, 2016) invariance of measurement across different groups. As a criterion for comparing different levels of invariance, −0.01 change in CFI was used, since the CFI change is regarded as a more reliable criterion than a significance of χ^2^ (French and Finch, 2006; Meade et al., 2008).

For the MCFAs we used social groups that were mentioned in previous studies as having different levels of SJ: gender groups, religious and non-religious people, and people with different socio-economic status (for review see, Jost, 2019). The respondents were divided into subgroups based on their answers to socio-demographic questions (for details on the number of people in each subgroup, see Table 1).

Table 6 shows the results of the MCFAs, which suggest that the general, political, and economic SJ scales demonstrated the full equivalence of factor structure, loadings, and item intercepts across the tested groups (in all cases δCFI < 0.01, indicating that models do not differ from one other). This result allows a reasonable comparison of the means in the indicated groups.

**Table 6:** The results of the MCFAs.

**Group**	**Model**	**χ2**	***df***	**RMSEA**	**95%CI RMSEA**	**SRMR**	**CFI**	**TLI**	**AIC**	**BIC**	**δχ2**	**δ** ***df***	**δCFI**
**GENERAL SYSTEM JUSTIFICATION**													
Gender	Configural	28.229	10	0.060	[0.040, 0.081]	0.016	0.989	0.978	19,267	19,414	10.929		
	Metric	36.845	14	0.057	[0.039, 0.076]	0.027	0.986	0.980	19,266	19,394	8.616	4	0.003
	Scalar	48.646	18	0.058	[0.041, 0.075]	0.032	0.981	0.979	19,270	19,378	11.801	4	0.005
Religiosity	Configural	23.966	10	0.059	[0.037, 0.082]	0.020	0.988	0.977	15,409	15,550	6.666		
	Metric	35.549	14	0.062	[0.042, 0.083]	0.042	0.982	0.974	15,413	15,535	11.583	4	0.006
	Scalar	48.389	18	0.065	[0.047, 0.084]	0.046	0.975	0.972	15,419	15,522	12.840	4	0.007
Socio-economic status	Configural	26.116	15	0.047	[0.021, 0.070]	0.017	0.993	0.986	19,215	19,436	8.816		
	Metric	34.274	23	0.038	[0.01, 0.060]	0.028	0.993	0.991	19,204	19,386	8.158	8	0.000
	Scalar	49.095	31	0.042	[0.020, 0.060]	0.033	0.989	0.989	19,204	19,346	14.821	8	0.004
**POLITICAL SYSTEM JUSTIFICATION**													
Gender	Configural	137.486	54	0.055	[0.046, 0.065]	0.018	0.982	0.977	34,454	34,719	31.05		
	Metric	150.413	62	0.053	[0.044, 0.062]	0.026	0.981	0.978	34,444	34,671	12.927	8	0.001
	Scalar	162.877	70	0.051	[0.042, 0.060]	0.027	0.980	0.980	34,438	34,625	12.464	8	0.001
Religiosity	Configural	127.239	54	0.058	[0.048, 0.069]	0.019	0.980	0.973	27,454	27,707	20.803		
	Metric	143.096	62	0.057	[0.047, 0.067]	0.034	0.978	0.974	27,450	27,666	15.857	8	0.002
	Scalar	161.745	70	0.057	[0.047, 0.067]	0.036	0.975	0.974	27,453	27,631	18.649	8	0.003
Socio-economic status	Configural	160.853	81	0.054	[0.044, 0.064]	0.020	0.983	0.978	34,388	34,786	54.417		
	Metric	181.256	97	0.051	[0.041, 0.061]	0.032	0.982	0.980	34,368	34,687	20.403	16	0.001
	Scalar	192.929	113	0.046	[0.036, 0.056]	0.033	0.983	0.984	34343	34584	11.673	16	0.001
**ECONOMIC SYSTEM JUSTIFICATION**													
Gender	Configural	39.8	18	0.049	[0.031, 0.067]	0.027	0.980	0.968	27,092	27,269	13.508		
	Metric	47.44	23	0.046	[0.029, 0.062]	0.034	0.978	0.972	27,088	27,240	7.64	5	0.002
	Scalar	57.986	28	0.046	[0.031, 0.061]	0.037	0.973	0.971	27,088	27,216	10.546	5	0.005
Religiosity	Configural	48.046	18	0.065	[0.045, 0.084]	0.034	0.967	0.945	21,647	21,816	21.754		
	Metric	55.879	23	0.060	[0.042, 0.078]	0.042	0.964	0.953	21,643	21,789	7.833	5	0.003
	Scalar	66.927	28	0.059	[0.042, 0.076]	0.046	0.957	0.954	21,644	21,766	11.048	5	0.007
Socio-economic status	Configural	44.599	27	0.044	[0.023, 0.063]	0.030	0.984	0.974	27,27	27,292	18.307		
	Metric	54.521	37	0.038	[0.016, 0.056]	0.036	0.984	0.981	27,014	27,231	9.922	10	0.000
	Scalar	67.556	47	0.036	[0.016, 0.053]	0.040	0.982	0.982	27,007	27,174	13.035	10	0.002
**GENDER SYSTEM JUSTIFICATION**													
Gender	Configural	25.408	10	0.055	[0.032, 0.079]	0.026	0.979	0.958	22, 100	22,248	3.251		
	Metric	47.431	14	0.069	[0.050, 0.088]	0.052	0.954	0.935	22,120	22,248	22.023	4	0.025
	Scalar	60.445	18	0.068	[0.051, 0.086]	0.056	0.942	0.936	22,124	22,233	13.014	4	0.012
Religiosity	Configural	23.909	10	0.059	[0.033, 0.085]	0.026	0.978	0.956	17,698	17,839	1.752		
	Metric	34.281	14	0.060	[0.038, 0.083]	0.046	0.968	0.954	17,702	17,824	10.372	4	0.010
	Scalar	45.730	18	0.062	[0.042, 0.082]	0.051	0.956	0.951	17,706	17,809	11.449	4	0.012
Socio-economic status	Configural	39.837	15	0.070	[0.046, 0.095]	0.029	0.968	0.936	22,105	22,326	17.68		
	Metric	48.846	23	0.058	[0.037, 0.078]	0.042	0.967	0.957	22,101	22,283	9.009	8	0.001
	Scalar	56.357	31	0.049	[0.030, 0.068]	0.044	0.968	0.969	22,092	22,234	7.511	8	0.001

In particular, men did not differ from women in justification of the social, political, and economic status quo [*t*_(1, 009)_ = −0.996, *p* = 0.319, *d* = −0.06; *t*_(1, 009)_ = −0.895, *p* = 0.370, *d* = 0.06; *t*_(1, 009)_ = 0.644, *p* = 0.519, *d* = 0.04, respectively]. Religious people more than non-religious people justify the status quo in social, *t*_(1, 009)_ = 2.77, *p* = 0.006, *d* = 0.21; political, *t*_(1009)_ = 5.72, *p* < 0.001, *d* = 0.44; and economic, *t*_(1, 009)_ = 4.28, *p* < 0.001, *d* = 0.32 relationships. Finally, high-status individuals more than low-status individuals justified the status quo in economic (*p* = 0.01), political (*p* < 0.001), and social relationships (*p* < 0.001) but did not differ in justification from those in the middle class (all *p* > 0.05).

More ambiguous results were obtained regarding the invariance of the gender SJ scale. For gender groups and people with different social status, items demonstrated different factor loadings, which may indicate that some additional factors may impact how different groups perceive the system of gender relationships. These factors can be related to the degree of a person's gender expectations and maintenance of gender roles. At the same time, when groups were divided according to religiosity, the gender SJ scale demonstrated full invariance.

#### Convergent and Discriminant Validity

To check the convergent and discriminant validity of the scales, a correlation analysis was carried out, the results of which are shown in Table 7. In agreement with previous data, the general SJ showed significant positive correlations with other SJ scales (*r* from 51 to 69) as well as with right-wing authoritarianism, social dominance orientation, and the just-world belief. Political SJ positively correlated with political self-efficacy and negatively with readiness to protest. The economic SJ scale correlated negatively with perceived economic inequality, the support for a base income concept, and redistribution of resources, while the gender SJ scale correlated positively with hostile and benevolent sexism. The correlations obtained confirm that SJ scales are positively related to other ideologies that support the status quo and negatively with initiatives and actions that are aimed at changing the existing system. These results are in line with the theoretical framework of SJT (for review see, Jost, 2019) and, consequently, confirm a convergent validity of the scales. Additionally, none of the SJ scales is associated with openness to experience, which is also consistent with previously data obtained (ibid) and confirms the discriminant validity of the scales.

**Table 7:** Correlational analysis results.

	**Mean**	**SD**	**a**	**1**	**2**	**3**	**4**	**5**	**6**	**7**	**8**	**9**	**10**	**11**	**12**	**13**	**14**	**15**
1. General SJ	3.69	1.83	0.89															
2. RWA	2.91	0.95	0.88	0.38***														
3. SDO	2.04	0.89	0.78	0.18***	0.22***													
4. JWB	3.53	1.05	0.80	0.40***	0.26***	0.13***												
5. OTE	3.69	0.62	0.80	−0.01	−0.03	−0.14***	0.07*											
6. Political SJ	4.15	2.16	0.95	0.69***	0.59***	0.19***	0.34***	−0.02										
7. PSE	2.32	1.13	0.89	0.63***	0.53***	0.18***	0.31***	−0.02	0.82***									
8. RTP	2.55	1.18	–	−0.22***	−0.22***	−0.04	−0.08*	0.10**	−0.34***	−0.27***								
9. Economic SJ	4.95	1.78	0.79	0.58***	0.54***	0.15***	0.31***	0.02	0.73***	0.62***	−0.19***							
10. PEI	4.96	1.01	–	−0.45***	−0.19***	−0.19***	−0.18***	0.07*	−0.45***	−0.45***	0.14***	−0.34***						
11. BI	5.13	1.95	–	−0.17***	0.04	−0.08**	−0.12***	0.02	−0.12***	−0.10**	0.03	−0.05	0.12***					
12. ED	4.48	1.96	–	−0.18***	0.13***	−0.09**	−0.16***	0.02	−0.13***	−0.10**	0.11***	−0.06	0.20***	0.44***				
13. Gender SJ	5.16	1.73	0.76	0.51***	0.39***	0.19***	0.36***	−0.04	0.52***	0.49***	−0.10**	0.53***	−0.32***	−0.06	−0.07*			
14. HS	3.94	1.50	0.87	0.07*	0.37***	0.24***	0.12***	−0.10**	0.18***	0.16***	−0.13***	0.19***	−0.06	0.09**	0.09**	0.34***		
15. BS	4.21	1.71	0.90	0.15***	0.50***	0.13***	0.16***	0.01	0.31***	0.28***	−0.11***	0.29***	−0.04	0.09**	0.17***	0.33***	0.40***	

*SJ, system justification; RWA, right-wing authoritarianism; SDO, social dominance orientation; JWB, just world belief; OTE, openness to experience; PSE, political self-efficacy; RTP, readiness to protest; PEI, perceived economic inequality; BI, base income support; ED, equal distribution support; HS, hostile sexism; BS, benevolent sexism*.

* *p < 0.05*,

** *p < 0.01*,

*** *p < 0.001*.

## General Discussion

The main aim of this paper was to adapt the SJ scales for the Russian socio-cultural context and to carry out a thorough psychometric analysis of them. The results obtained suggest that none of the original SJ scales work properly in Russia and demonstrate weak fit indices. However, adapted scales with additional items relating to the specific socio-cultural context exhibit fits that are a good to excellent. Unlike most other adaptations of the SJ scales, we not only tested the consistency of the adapted scales, but also demonstrated their invariance for different groups and confirmed their convergent and discriminant validity. This gives us reason to consider them as an effective tool for measuring system justification in various types of social relations in Russia.

The process of adapting the SJ scales for Russia also facilitates further reflection on the role of cultural characteristics in justification of the status quo. The culture and history of one's country frame the specific understanding of what the system is and how it should operate. For example, China is characterized by a long history of meritocratic culture associated with Confucianism. This “settles” the low status of certain groups and prevents widespread social mobility, which affects how people perceive and justify the system (Li et al., 2020). In contrast, the USA, where the system justification theory originated, has for most of its history been defined by democracy and capitalism.

In the case of Russia, 30 years ago the country underwent one of the most severe communist to capitalist transformations in recent history. Over the last century, 70 years of the USSR's system, with its planned economy, one-party politics, and socialist society resulted in the formation of the Russian Federation with a capitalist economy, multi-party system, and democratic society. Such a history could form a unique consciousness, which is different from legitimizing myths in other developed countries. For example, in Russia, according to polls, more than 60% of the population expects the state to show paternalistic concern about the quality of life (Levada Center, 2020e). This may have led to the fact that the items relating to the economic SJ proposed by Jost and Thompson (2000) appeared unacceptable for the Russian sample.

The case study of SJ in Russia may add new nuances to this explanation. Russia is the successor of the Soviet Union and therefore connected with a greater extent to the Soviet past. Political scientist Vladimir Gel'man discussed the paradoxical passive behavior of Russian citizens with low levels of political legitimacy beliefs and high electoral support for the current system, and proposed the concept of legacy of the Soviet past—at the end of the Soviet era it was easier to choose the “exit” strategy and ignore the conventional political and social system than to fight it (Gel'man, 2010). Indeed, polls show that, combined with low trust in various political and social institutions (Levada Center, 2020f), most Russians are not ready to participate in the political process (Levada Center, 2019). Instead, they assume that politics is difficult to understand by ordinary people and that it is hard to change anything. At the same time, more than 60% of Russians regret the collapse of the USSR and believe that it could have been avoided (Levada Center, 2018a).

In other words, in the post-Soviet era, the low level of SJ can be associated not only with general disappointment in any socio-political systems, but also with specific attitudes toward the communist past.

An alternative explanation for the low level of SJ may be related to the current socio-political context and discourse. First, the data were collected during spring-autumn 2020 when the COVID-19 pandemic was spreading all over the world. This could have led to decreasing SJ if citizens perceived a governmental response to the pandemic as ineffective. Second, in the spring of 2020, a referendum about proposed changes to the constitution was held in Russia. The discussion about the amendments increased the differentiation of people. By the summer, public opinion polls showed that 43% of Russians believed that things in Russia were going in the right direction, and 41% that things were going in the wrong direction (Levada Center, 2020a). Both key events could impact the level of SJ.

Undoubtedly, additional research is needed to better understand the specifics of justifying the system in Russia and in various post-communist countries. Our results only indicate the presence of cultural specificity, a deeper understanding of which is still ahead.

## Data Availability Statement

The datasets presented in this study can be found in online repositories. Open Science Framework https://osf.io/3wxge/?view_only=353fa3a12dca45a18c68cc003bd36eb5.

## Ethics Statement

Ethical review and approval was not required for the study on human participants in accordance with the local legislation and institutional requirements. The patients/participants provided their written informed consent to participate in this study.

## Author Contributions

EA: conceptualization, methodology, formal analysis, writing, supervision, and funding acquisition. IS: conceptualization, methodology, and writing. AI: formal analysis and writing. All authors contributed to the article and approved the submitted version.

## Funding

This work was supported by Russian Science Foundation (grant #20-18-00142).

## Conflict of Interest

The authors declare that the research was conducted in the absence of any commercial or financial relationships that could be construed as a potential conflict of interest.

## Publisher's Note

All claims expressed in this article are solely those of the authors and do not necessarily represent those of their affiliated organizations, or those of the publisher, the editors and the reviewers. Any product that may be evaluated in this article, or claim that may be made by its manufacturer, is not guaranteed or endorsed by the publisher.
