# Supplementary material for: How Do Russians Perceive and Justify the Status Quo: Insights From Adapting the System Justification Scales
Source: Front Psychol. 2021 Oct 21;12:717838. doi: 10.3389/fpsyg.2021.717838 (PMC8566909; doi:10.3389/fpsyg.2021.717838)
Supplement: Supplementary file 1 [file Data_Sheet_1.docx]

**Appendix**

|  | **In English** | **In Russian** |
| --- | --- | --- |
| **№** | *General system justification* |  |
|  | In Russia today, | Сегодня в России |
| 1 | … life in general is fair. | … в большинстве случаев жизнь устроена справедливо |
| 2 | … the life of most people does not require significant changes | … жизнь большинства людей не требует существенных изменений |
| 3 | … most of the authorities’ decisions are aimed at the good of the people. | … **большинство решений власти направлены на благо народа** |
| 4 | … most people can achieve their desired standard of living | … **большинство людей может достичь желаемого уровня жизни** |
| 5 | … life of most people become better | … **у большинства людей жизнь стала лучше** |
| 6 | … most people should be happy with what they have | … **большинство людей должны быть довольны тем, что имеют** |
| 7 | … the lives of most people are changing in the right way | … **жизнь большинства людей меняется в правильном направлении** |
| 8 | … life in most cases corresponds to our history and traditions | … жизнь в большинстве случаев соответствует нашей истории и традициям |
| 9 | … the main task is to maintain stability | … главная задача - это сохранение стабильности |
| 10 | … the state has its own special way of development | … у государства свой особенный пусть развития |
| 11 | … most people can achieve a lot if they have the will | … большинство людей может многого добиться, если будет иметь желание |
|  | *Political system justification* |  |
| 1 | In Russia today, the political system is the best in the world. | **Сегодня в России власть - одна из лучших в мире** |
| 2 | In Russia today, despite some problems the authorities provide protection against global shocks in most cases | **Сегодня в России власть, несмотря на отдельные проблемы, в большинстве случаев обеспечивает защиту от глобальных потрясений** |
| 3 | In Russia today, the authorities deservedly have the support of the majority | **Сегодня в России власть заслуженно пользуется поддержкой большинства** |
| 4 | In Russia today, the authorities in most cases acts in the interests of the people. | **Сегодня в России власть в большинстве случаев действует в интересах народа** |
| 5 | In Russia today, having one party that is supported by the majority ensures stability | **Сегодня в России наличие одной партии, которую поддерживает большинство, обеспечивает стабильность** |
| 6 | In Russia today, the decisions of the authorities can be trusted in most cases | **Сегодня в России в большинстве случаев решениям власти можно доверять** |
| 7 | In Russia today, stable authorities are the basis of the country’s successful development | **Сегодня в России стабильная власть - это основа успешного развития** |
| 8 | In Russia today, problems come mostly from local authorities, not from the government in general | Сегодня в России большинство проблем связано с ошибками мелких чиновников |
| 9 | In Russia today the change of political system will do no good. | **Сегодня в России смена власти только навредит** |
| 10 | In Russia today, authorities share national resources with people | Сегодня в России власть делится с народом национальными ресурсами |
| 11 | In Russia today, authorities are close to the people | Сегодня в России власть близка к народу |
| 12 | In Russia today, the decisions of authorities are in line with our history and traditions | **Сегодня в России власть в большинстве случаев принимает решения в соответствии с нашей историей и традициями** |
| 13 | In Russia today, any good intent from the government could end up as nothing due to local authorities | Сегодня в России любые благие намерения власти могут испортить местные чиновники |
|  | *Gender system justification* |  |
| 1 | In Russia today, the husband must earn money while the wife must take care of the children and the house. | Сегодня в России муж в первую очередь должен зарабатывать деньги, а жена - заниматься детьми и домом |
| 2 | In Russia today, the preservation of gender roles is determined by our history and traditions | Сегодня в России мужские и женские роли определены нашей историей и традициями |
| 3 | In Russia today, women have better live than in many other countries | **Сегодня в России женщинам живется лучше, чем во многих других странах** |
| 4 | In Russia today, the restrictions for women in the choice of "male" professions (e.g., a miner or a machinist) are dictated by concern for their health and safety | Сегодня в России ограничения для женщин в выборе "мужских" профессий (например, шахтер или машинист) продиктованы заботой об их здоровье и безопасности |
| 5 | In Russia today, men and women have equal career opportunities. | **Сегодня в России мужчины и женщины имеют равные карьерные возможности** |
| 6 | In Russia today, most men and women in general should be satisfied with what they have. | **Сегодня в России большинство мужчин и женщин должны быть довольны тем, что имеют** |
| 7 | In Russia today, the relationships between men and women are what they are supposed to be | **Сегодня в России взаимоотношения между мужчинами и женщинами такие, какими они и должны быть** |
| 8 | In Russia today, it is good to be a woman. | **Сегодня в России хорошо быть женщиной** |
| 9 | In Russia today, femininity in women and masculinity in men give them an advantage in life. | **Сегодня в России женственность у женщин и мужественность у мужчин дают им преимущество в жизни** |
| 10 | In Russia today, stereotypes about men and women are true | Сегодня в России стереотипы о мужчинах и женщинах соответствуют действительности |
|  | *Economic system justification* |  |
| 1 | In Russia today, salaries are fair and adequate. | Сегодня в России разрыв в доходах между богатыми и бедными значительно меньше, чем в других странах |
| 2 | In Russia today, it is impossible to reduce the number of poor people, no matter what the authorities do | Сегодня в России невозможно уменьшить количество бедных людей, что бы ни предпринимали власти |
| 3 | In Russia today, it is big business that provides real support for the economy. | **Сегодня в России крупный бизнес - это то, что реально поддерживает экономику** |
| 4 | In Russia today, the income gap is in line with our history and traditions | Сегодня в России разрыв в доходах между богатыми и бедными определен нашей историей и традициями |
| 5 | In Russia today, free medical care and education are a big advantage over other countries | **Сегодня в России бесплатное образование и медицина - это то, что делает ее лучше многих других стран** |
| 6 | In Russia today, sanctions from other countries essentially have no impact on the economy | **Сегодня в России санкции других стран не влияют существенно на экономику** |
| 7 | In Russia today, government control of resources is necessary to support the economy. | **Сегодня в России государственный контроль над ресурсами - необходимая мера для поддержания экономики** |
| 8 | In Russia today, our groceries are not inferior in quality to those from foreign countries | **Сегодня в России продукты питания российского производства в большинстве случаев не уступают зарубежным** |
| 9 | In Russia today, the economic situation is no worse than in many other countries. | **Сегодня в России экономическая ситуация ничем не хуже, чем во многих других странах** |
| 10 | In Russia today, will always be poor people, because there will never be enough jobs for everybody. | Сегодня в России бедность неизбежна, потому что рабочих мест все равно не хватит на всех |
| 11 | In Russia today, taxes are significantly lower than in other countries. | Сегодня в России налоги меньше, чем во многих других странах |

Note: Highlighted judgments were included in the final versions of the scales
